# Deep convolutional neural networks for annotating gene expression patterns in the mouse brain

**DOI:** 10.1186/s12859-015-0553-9

**Published:** 2015-05-07

**Authors:** Tao Zeng, Rongjian Li, Ravi Mukkamala, Jieping Ye, Shuiwang Ji

**Affiliations:** 1 Department of Computer Science, Old Dominion University, Norfolk, 23529 VA USA; 2 Department of Computational Medicine and Bioinformatics, University of Michigan, Ann Arbor, 48109 MI USA; 3 Department of Electrical Engineering and Computer Science, University of Michigan, Ann Arbor, 48109 MI USA

## Abstract

**Background:**

Profiling gene expression in brain structures at various spatial and temporal scales is essential to understanding how genes regulate the development of brain structures. The Allen Developing Mouse Brain Atlas provides high-resolution 3-D *in situ* hybridization (ISH) gene expression patterns in multiple developing stages of the mouse brain. Currently, the ISH images are annotated with anatomical terms manually. In this paper, we propose a computational approach to annotate gene expression pattern images in the mouse brain at various structural levels over the course of development.

**Results:**

We applied deep convolutional neural network that was trained on a large set of natural images to extract features from the ISH images of developing mouse brain. As a baseline representation, we applied invariant image feature descriptors to capture local statistics from ISH images and used the bag-of-words approach to build image-level representations. Both types of features from multiple ISH image sections of the entire brain were then combined to build 3-D, brain-wide gene expression representations. We employed regularized learning methods for discriminating gene expression patterns in different brain structures. Results show that our approach of using convolutional model as feature extractors achieved superior performance in annotating gene expression patterns at multiple levels of brain structures throughout four developing ages. Overall, we achieved average AUC of 0.894 ± 0.014, as compared with 0.820 ± 0.046 yielded by the bag-of-words approach.

**Conclusions:**

Deep convolutional neural network model trained on natural image sets and applied to gene expression pattern annotation tasks yielded superior performance, demonstrating its transfer learning property is applicable to such biological image sets.

## Background

Accurate spatiotemporal control of gene expression drives the development of brain structure and function. The development of individual structures and the corresponding neuronal connectivity is the consequence of gene expression patterns that change spatially and temporally. Therefore, accurate characterization of the patterns and levels of gene expression, such as local expression gradient patterns and levels in various brain structures, is essential to understanding brain development.

The Allen Developing Mouse Brain Atlas (ADA) consists of 3-D, cellular resolution *in situ* hybridization (ISH) expression patterns of approximately 2,000 genes in sagittal plane across multiple developmental stages [1,2]. In addition, the Allen Developing Mouse Brain Reference Atlas (ARA) provides the brain structural ontology based on developmental neuroanatomy. This makes it possible to characterize the gene expression signals as patterns and levels corresponding to the brain structures at multiple hierarchical levels. Such annotations, which were currently performed manually using the expertise of neuroscientists, enable neuroscientists to explore the intrinsic mechanism as to how genes regulate the development of brain at fine structure levels. However, manually annotating gene expressions over an enormous number of ISH images is labor-intensive and may result in inconsistence among different experts [3].

In this study, we consider the approach of automated image computing as a way to automate such task [4,5]. To yield discriminative features, a common approach is to compute local descriptors on a large number of image patches and then build global representations by using various approaches such as the bag-of-words method. Such techniques have yielded promising performance on various natural and biological image classification tasks [5].

In contrast, deep learning models are a class of multi-layer systems that can be trained end-to-end to learn hierarchical features from raw data. As one of the common deep learning models, deep convolutional neural networks (CNN) have gained increasing attention due to their superior performance on various tasks [6-8]. However, a large number of labeled examples are required to train the parameters in CNN. To overcome this limitation, recent studies used the ImageNet data, an image data set with thousands of categories and millions of labeled natural images, to train a CNN model. The learned model was then used as feature extractors for other data sets. Such transfer learning approach yielded promising performance on a wide variety of recognition tasks [9-13]. These studies show that CNN can be used for transfer learning, where the network is trained on one data set and used as feature extractor on other data sets.

In this work, we propose to use CNN for knowledge transfer from natural images to ISH images. We explored whether the transfer learning property of CNN observed on natural images could be generalized to biological images. Specifically, we used trained model from OverFeat as feature extractors on ISH images. The resulting features were subsequently used to train and validate our machine learning method for annotating gene expression patterns. We compared our results with those yielded by the bag-of-word method. Results show that our approach of using convolutional model as feature extractors achieved superior performance on the tasks of annotating gene expression patterns at multiple levels of brain structures throughout four developing ages. We achieved an overall average AUC of 0.894 ± 0.014, as compared with 0.820 ± 0.046 yielded by the bag-of-words approach.

## Methods

### Allen developing mouse brain atlas

The Allen Developing Mouse Brain Atlas (ADA) provides a framework for exploring spatiotemporal dynamics of gene expression over the course of mouse brain development [1]. ISH image data are available for about 2,000 genes in the sagittal section across seven developing stages. For each gene, ISH was applied to multiple sections of the brain to detect a specific gene expression covering the entire brain. These ISH images are subsequently processed by an informatics data processing pipeline to generate grid-level voxel data.

**Figure 1:**
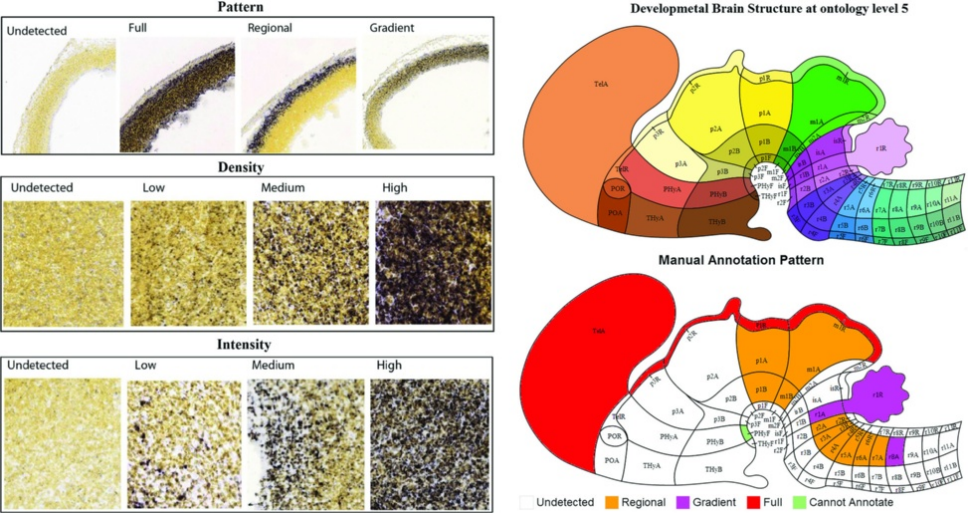
Illustration of the manual annotations. The figure on the left illustrates the three different metrics. The top-right figure shows the brain structure ontology at level 5. The bottom-right figure denotes the corresponding manual annotations. The figures were reproduced from [3] with permission.

Characterizing gene expression patterns, such as local expression gradients and levels, in brain structures at various levels throughout multiple developing stages allows neuroscientists to explore the crucial temporal and spatial events during development [14]. To characterize structural level gene expression, a reference atlas was created to segment the brain into structures through all developing stages. The subsequent annotation of gene expression patterns and levels of intensity and density at structural levels was then determined manually using the expertise of neuroscientists [3]. Specifically, the experts first attempted to identify whether a given gene expression was detectable in a specified structure. They subsequently annotated this gene expression using pattern, intensity and density metrics if it is detectable. Otherwise, the gene was labeled as “undetected” for all three metrics. The pattern metric was scored as full, regional, and gradient, whereas density and intensity metrics were scored as low, median and high, respectively (Figure 1). Currently, manual annotations have been generated for four (E11.5, E13.5, E15.5, and E18.5) out of the seven developing stages by Allen Institute for Brain Science.

### A baseline ISH image representation

In this work, we consider the automated annotation of gene expression patterns. This requires a computational representation to encode the expression patterns in the ISH images. We first considered a baseline approach based on the bag-of-words representation that has been widely used in modeling natural and biological images [5,15-20]. We then proposed to employ a deep convolutional feature extractor in the following section, and this new deep representation yielded better performance in our experiments.

To obtain robust representations that are invariant to various distortions on the images, scale-invariant feature transform (SIFT) descriptors were applied on local patches of ISH images that were down-sampled by a factor of 4 to reduce the computational cost [21]. We applied dense SIFT feature descriptors on the ISH images implemented in the VLFeat software package [22]. This generated approximately 20,000 SIFT feature vectors from each ISH image section.

The bag-of-words approach requires a visual codebook for vector quantization. To this end, we randomly sampled the nonzero descriptors for each image to obtain a descriptor pool of size 100,000. The *K*-means algorithm was then applied to cluster the SIFT descriptors into 500 clusters in this pool, and the resulting cluster centers were considered as visual words in the codebook. We repeated the *K*-means algorithm multiple times with random initializations and used the one with the smallest within-cluster distance, since initialization may impact the results of *K*-means algorithm. In our approach, the SIFT features were extracted from the ISH images using three different scales. Accordingly, we constructed three separate codebooks for all images from a single development stage and counted number of occurrences of each visual word, resulting in multiple bags of visual words for each image. We concatenated these representations to form a single representation for the image. In addition, we used an extra dimension to account for the number of zero descriptors for each scale.

To represent gene expression patterns covering the entire 3-D brain, we divided the brain sagittally into seven intervals. Each ISH image section was assigned to one of the seven intervals based on its spatial location. The corresponding bag-of-words representations of ISH images assigned to the same interval were averaged to reflect regional sagittal gene expression. The global representation was built by concatenating the seven regional bag-of-words vectors. As a result, the final global representation for each gene covering entire brain is a feature vector with size of 10521 (501×3×7).

### Deep convolutional neural networks for feature extraction

Deep learning models are based on the idea that representations of observed data are the results of hierarchical abstraction at many different levels [6,7]. Hence, such model can learn a hierarchy of features by building high-level features from low-level ones. Convolutional neural networks (CNN) are a class of deep models that were inspired by information processing in the brain. CNN mimics the receptive field of biological neuron, and each unit in CNN receives local inputs from lower level. CNN also uses replicated weight matrix for all units in the same feature map to compute the same feature from all locations on the inputs [6,7].

CNN requires a large number of training samples in order to achieve competitive performance. To overcome such limitation, recent studies adapted the approach of transferring knowledge from one image data set to another, yielding superior performance on a wide variety of object recognition tasks [9-12].

In this paper, we explored whether the well-performed feature generalization achieved on natural images could also yield similar performance when applied to biological images. Specifically, we used the OverFeat model [23] that was trained on the ImageNet data as feature extractors for the ISH images of developing mouse brain. OverFeat provides two pre-trained models known as the “accurate” and “fast” models. The accurate model yields larger feature vectors. We used the fast model in the experiments to reduce the computational cost.

**Figure 2:**
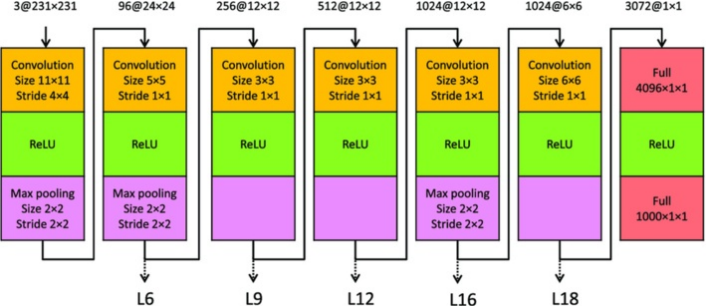
The architecture of the OverFeat “fast” model. This model consists of 22 layers. Each column represents one stage consisting of convolution, rectified linear units (ReLU), and optionally max-pooling. We extracted features from layers 6, 9, 12, 16, and 18.

The OverFeat architecture contains many stages of layers, and each stage consists of convolution, rectified linear units, and optionally max-pooling layers. Specially, the accurate model contains 7 stages consisting of 22 layers as shown in Figure 2. Note that we omitted the last soft-max layer and grouped the last two full connection layers into one stage for better visualization. The outputs of layers within each stage are similar. We have experimentally verified that the features extracted from different layers in the same stage usually lead to the similar results. We thus extracted features only from the last layer in each stage. That is, we used the ISH images as inputs to the network and extracted features at layers 6, 9, 12, 16, and 18 for each ISH image. The numbers of feature maps in these layers are 256, 512, 1024, 1024, and 3072, respectively. The corresponding sizes of feature maps are 12 × 12, 12 × 12, 12 × 12, 6 × 6, and 1 × 1. We flattened all feature maps into vectors and concatenated them into a single feature vector for each layer. As a result, the corresponding sizes of feature vectors for those layers are 36864, 73728, 147456, 36864 and 3072, respectively.

**Figure 3:**
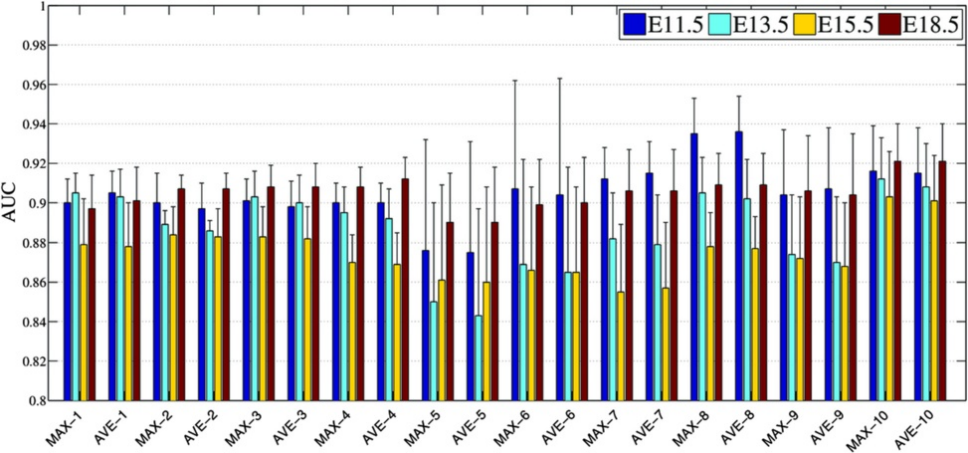
Performance achieved by element-wide average across all feature vectors of section images (AVE) versus those of element-wide maximum (MAX). Features were extracted by OverFeat at layer 12. The numbers in the x-axial label indicate the corresponding anatomical ontology level of the mouse brain.

As each ISH experiment generated 15-20 sagittal image sections, to build a feature vector that represents the gene expression pattern covering the entire brain, we extracted features for each section separately and computed element-wide maximum across feature vectors from all sections of the same experiment. We have also used element-wide average to combine the vectors, and this yielded slightly lower performance as can be seen from Figure 3. The pipeline for feature extraction can be summarized as follows: First, we resized all images to 231 × 231 as required by the OverFeat model. We then extracted feature vectors from each network layer for each ISH image. Finally, we computed element-wide maximum across feature vectors of all section images of a gene expression. This global feature vector represents the global gene expression covering the entire brain and was used to perform gene expression pattern annotation.

### Gene expression pattern annotation

The ISH images were manually annotated with multiple expression patterns and levels of intensity and density for each individual brain structure across multiple ontology levels, ranging from the lowest level 10 to the highest level 1. Using the feature vectors generated from our method, our task is to automatically annotate gene expression patterns for a given developing stage. We trained a classification model to annotate gene expression patterns for each brain structure. There are 3, 6, 19, 14, 81, 46, 123, 40, 307, 432 structures from level 1 to 10, respectively. Hence, the total number of classifiers is 1071.

Given a set of training samples $\{\textbf {x}_{i},y_{i}\}_{i=1}^{n}$, where $\textbf {x}_{i}\in \mathbb {R}^{p}$ denotes the input feature vector, and *y*_*i*_∈{−1,1} denotes the corresponding output label. In this work, **x**_*i*_ represents the bag-of-words or deep convolutional feature vector, and *y*_*i*_ encodes the annotation of gene expression for a given brain structure. We employed the following regularized learning formulation for classification:

(1) $$ \min_{\textbf{w}} \sum_{i=1}^{n} L(\textbf{w}^{T} \textbf{x}_{i}+b,y_{i}) + \lambda \Omega(\textbf{w}),  $$

where $\textbf {w}\in \mathbb {R}^{p}$ and $b\in \mathbb {R}$ denote the model weight vector and bias term, respectively, *Ω*(**w**) denotes the regularization term, and *λ* is the regularization parameter. In this study, we employed the logistic regression loss function and the *ℓ*_2_-norm regularization *Ω*(**w**)=∥**w**∥_2_ as this model has been shown to yield competitive performance in classification tasks.

## Results and discussion

### Experimental setup

To train and evaluate our methods, we built four data sets, one for each developing stage. Each data set consists of global gene expression feature vectors corresponding to about 2,000 genes. Manual annotation of the Allen Developing Mouse Brain Atlas was performed by neuroanatomists at Allen Institute for Brain Science. Three different metrics (pattern, intensity, and density) were used to characterize different aspects of gene expression. In addition, the manual annotation was performed at multiple levels of the ontology hierarchy [3] as depicted in Section “Methods”.

**Figure 4:**
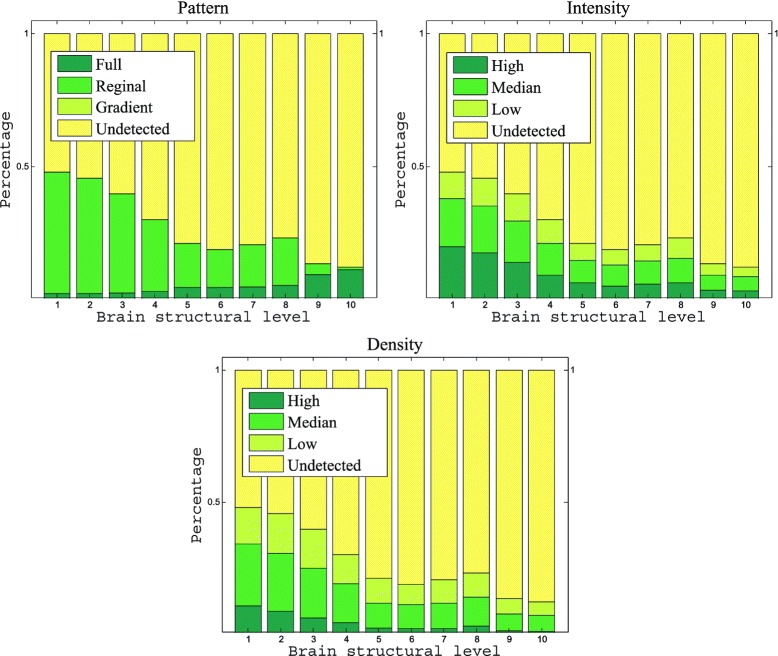
Percentage of images in each category of pattern, density, and intensity of gene expression across four developmental stages and ten ontology levels.

Figure 4 shows the statistics of data distribution at each of the pattern and level categories. We observed that the class “undetected” dominated all metrics of manual annotation. To alleviate this class imbalance problem, we simplified the classification tasks to a set of binary-class problems, where one class corresponds to the undetected category, and the other class includes all remaining categories. In addition, training and test samples were selected by maximizing the class balance. That is, at a given ontology level, we randomly selected training samples from the data set and checked whether the ratios between two classes among all structures were above a certain threshold. We repeated this process for a maximum of 5,000 times and then decreased the threshold if the ratio was not satisfied. Thus, the final thresholds are different for different data sets. By converting the annotation tasks into a two-class problem, the three metrics (pattern, intensity, and density) resulted in the same set of classification tasks. We thus focused on the pattern metric in our experiments.

To compare our proposed image representations with other methods, we also obtained the grid-level voxel features generated by the Informatics Processing Pipeline of Allen Institute for Brain Science. Given the training set and test set, the goal of our tasks is to annotate gene expression for all brain structures at a specified ontology level. We partitioned the entire data set into training and test sets so that 2/3 of the data were in the training set, and the remaining 1/3 were in the test set. For all different features, the same training and test sets were used. For each annotation task, we used the area under the ROC curve (AUC) as the performance measure. We reported the overall AUC values for annotation tasks on all brain structures at each given ontology level.

### Performance of automated annotation

**Figure 5:**
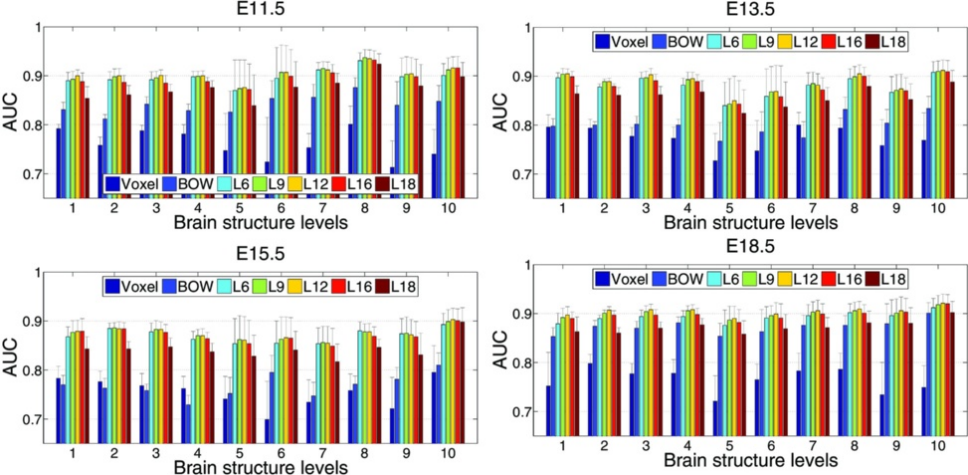
Performance comparisons of different features. Each figure shows the annotation performance for one developing stage. “BOW” and “Voxel” denote the performance achieved by the bag-of-words and grid-voxel level data, respectively. “Lx” denotes the performance of deep convolutional features, where “x” indicates the network layer from which the features were extracted.

**Figure 6:**
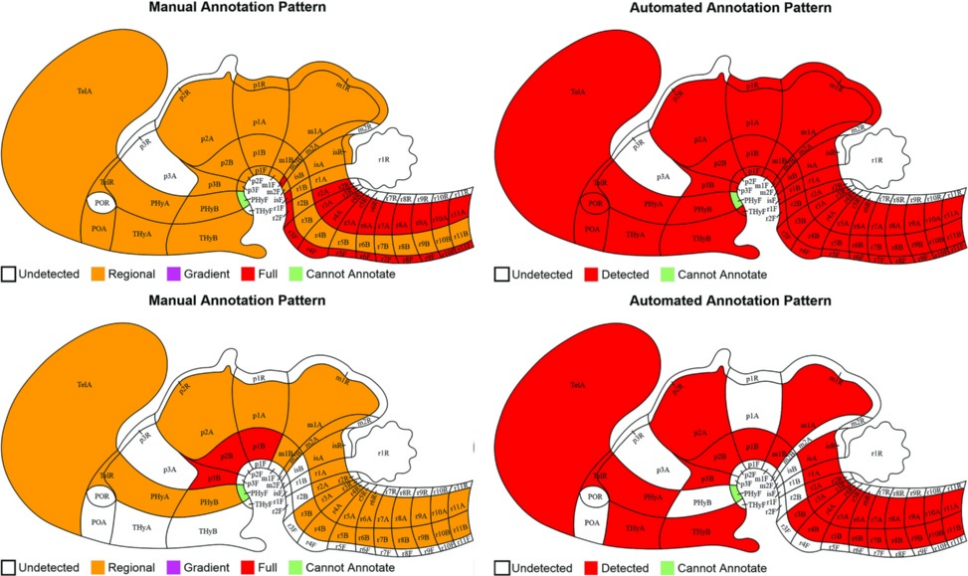
Examples of gene expression annotation at anatomical ontology level 5. Top row figures illustrate the expression pattern for the gene Fxyd6 at developing stage E18.5, and bottom row shows the gene Tnc at developing stage E15.5. The left column figures are manual annotation, and the right column figures illustrate the corresponding automated annotation by our proposed approach with features extracted at layer 12 of the deep convolution model.

We performed the tasks of gene expression annotation for all brain structures at various ontology levels ranging from 1 to 10. Due to the high cost of manual annotations, ground truth data is only available for four embryonic developing stages. We thus used data sets from developing stages E11.5, E13.5, E15.5, and E18.5. The detailed performance achieved by different methods are compared and reported in Table 1 and Figure 5. To illustrate the objective of automated annotation, the manual annotations generated at ontology level 5 for some sample genes were compared with those of automated annotations using features extracted from OverFeat layer 12 in Figure 6.

**Table 1:** **Overall AUC values achieved using different features across all ontology levels**

	**Voxel**	**BOW**	**L6**	**L9**	**L12**	**L16**	**L18**
**E11.5**	0.759	0.841	0.898	0.903	0.905	0.898	0.876
**E13.5**	0.768	0.800	0.880	0.886	0.889	0.881	0.859
**E15.5**	0.754	0.768	0.870	0.875	0.876	0.871	0.840
**E18.5**	0.764	0.873	0.893	0.901	0.905	0.898	0.873
**Overall**	0.761	0.820	0.885	0.891	**0.894**	0.888	0.862

“BOW” and “Voxel” denote the performance achieved by the bag-of-words and grid-voxel level data, respectively. “Lx” denotes the performance of deep convolutional features, where “x” indicates the network layer from which the features were extracted. Note that L12 achieved the highest overall AUC in comparison with those achieved by other layers. BOLD- L12 achieved the highest overall AUC in comparison with those achieved by other layers.

In comparison with grid-level features, the BOW representation achieved higher performance. Note that the BOW methods have been successfully applied to biological image applications in the past. However, few studies have used this approach for the processing of 3-D gene expression data sets to characterize gene expressions at fine anatomic brain structure levels.

**Figure 7:**
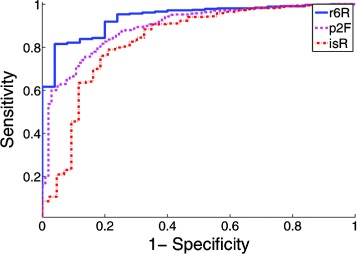
ROC curves for brain structures r6R, p2F and isR corresponding to the highest, median and the lowest AUC achieved among all brain structures at ontology level 5.

For the deep convolutional network features, we can observe from Table 1 and Figure 5 that features extracted from layer 12 achieved the best overall performance. In addition, the features of all 5 layers outperformed the bag-of-words as well as grid-level features at each of 4 developing stages. To examine the performance of individual brain structures, we showed the ROC curves for three brain structures at ontology level 5 with the highest, median and the lowest AUC values in Figure 7.

### Comparison between bag-of-words and convolutional features

**Figure 8:**
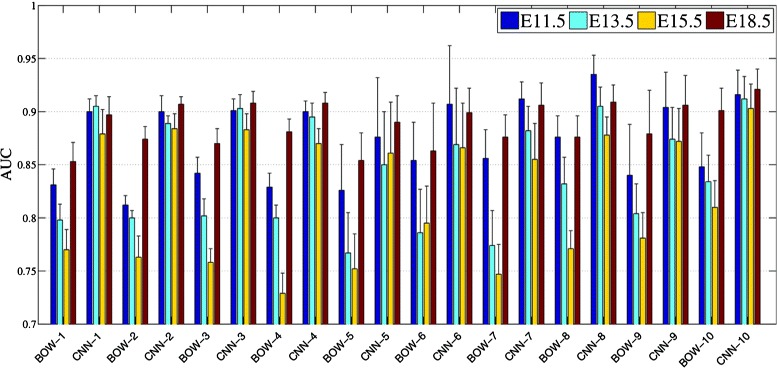
Performance achieved using features generated by the bag-of-words (BOW) and layer 12 of the OverFeat network for predicting gene expression at the brain structures of multiple ontology levels. The numbers in the x-axial label indicate the corresponding anatomical ontology level of the mouse brain.

Our results showed that, while the baseline BOW representations yielded higher performance than the grid-level voxel data, the convolution model achieved the best performance over all developing stages. Despite the fact that the performance of the BOW representations increases in stage E18.5, it is still lower than the performance by the convolutional model. In addition, it can be seen from Figure 8 that, although the performance of the BOW representations correlates with those achieved by the deep convolutional features across four developing stages, the BOW approach showed a larger variation in terms of extracting discriminative features across different stages. This indicates that the BOW representations are less robust to variation in the data sets as compared to the convolutional feature extractors. Overall, deep convolution models that were pre-trained by natural images exhibited impressive feature generalization power when applied to these biological image sets, yielding discriminative features superior to those extracted by the BOW methods.

### Comparison among different developing stages

We noticed that the relative performance of different methods differs across different developing stages. To ease the comparison, we grouped the results of different developing stages belonging to the same structural ontology level together in Figure 8. It can be observed that, for both the bag-of-words and convolutional feature representations, features obtained from stage E13.5 and E15.5 are less discriminative, yielding lower AUC for gene expression annotation tasks than those obtained from stage E11.5 and E18.5. Overall, annotation tasks in stage E18.5 achieved the highest AUC followed by stage E11.5. Those tasks in stage E15.5 yielded the lowest performance among all stages.

We noted that the ISH images provided by the Allen Developing Mouse Brain Atlas contain the whole embryo from stage E11.5 to stage E15.5. In contrast, the ISH images in stage E18.5 contain only the brain. Thus, we suspect that such image differences might be a factor that resulted in the performance difference achieved by the bag-of-words and convolutional representations in stage E18.5 as compared to other three stages.

### Gene expression patterns are best represented in the intermediate layers of CNN

The deep learning model is constructed in such way that a hierarchical feature representation is formed from low level to high level as the depth of network increases. When such networks are trained with natural images such as the ImageNet data set, the feature representations in the lower layers are expected to be generic features such as edge and corner detectors. In contrast, the features in higher layers are expected to represent objects specific to the training set. Hence, for the task of natural object recognition, the features extracted from higher levels usually yielded better discriminative power [10]. In our experiments, the discriminative power increases from layer 6 to layer 12, and then drops afterwards as the depth of network increases. This indicates that gene expression features are best represented in the intermediate layers of CNN that was trained on natural image sets. This might be explained by the fact that the high-level features mainly capture data set specific object information such as the natural objects from the ImageNet data set. Whereas, the task of identifying gene expression patterns of brain structures from ISH images is based on small portions of ISH images, and our current study only identified gene expression as either detected or undetected. Such tasks are likely to rely on texture-like information. Such type of local and texture-like information is usually represented in the intermediate layers of networks.

This observation raised an interesting point. That is, as the number of annotated ISH images increases for different model organisms, such as fruit fly, worm, fish, and mouse, we may be able to train a CNN using these images. We anticipate that the CNN trained with ISH images would achieve better performance than that trained with natural images.

### Functional annotation of genes

Inspired by the results that our approach of employing CNN outperformed the bag-of-words approach on the task of annotating gene expression patterns, we expand the application of CNN to the tasks of gene ontology functional annotation to show the generalization power of CNN pre-trained on natural images. In [17] such annotation was achieved using the bag-of-words approach on adult mouse brain. Similar to the approach described in Section “Methods”, we extracted features using the bag-of-words and CNN methods on adult mouse ISH image sets. We then compared the performance on the task of gene ontology annotation using these two types of features over 100 terms with the highest numbers of positive annotations. Our results show that CNN could achieve an average AUC of 0.5966±0.0294 over 100 terms. In comparison, the bag-of-words features yielded an average AUC of 0.5746±0.0240 (p <1.16×10^−13^, Wilcoxon signed rank tests). These results demonstrated that the features extracted from CNN are robust and can be used in different annotation tasks.

## Conclusion

In this study, we proposed methods for annotating the mouse brain gene expression patterns automatically. Altogether, the results showed that advanced computer vision and machine learning techniques we used are capable of achieving high accuracy. In addition, gene expression annotation can be achieved at various levels of brain structures without providing explicit spatial information. This is likely due to the robust properties of SIFT and deep convolution neural networks in producing invariance features. More interestingly, CNN model learned on natural image sets and applied to gene expression annotation tasks yielded superior performance, demonstrating its transfer learning property is applicable to such biological image sets.

Due to the high cost of manual annotation, expert annotation data are only available for four out of the seven developing stages. We will explore transfer learning techniques to predict the annotations in the other three stages by leveraging the knowledge in the currently annotated four stages. Due to the class imbalance problem in data set and the tremendous amount of high-resolution ISH images, we employed a binary-classification scheme in this work. However, the other gene expression levels and patterns would be more informative as they provide detailed information about gene expression of brain structures. We will explore advanced techniques to overcome the class imbalance problem and formulate the annotation problem into a set of multi-class classification tasks in the future.
